# Trends in total and cause-specific mortality by marital status among elderly Norwegian men and women

**DOI:** 10.1186/1471-2458-11-537

**Published:** 2011-07-06

**Authors:** Kjersti Norgård Berntsen

**Affiliations:** 1 Department of Health Management and Health Economics, University of Oslo, PO Box 1089, 0317 Oslo, Norway

## Abstract

**Background:**

Previous research has shown large and increasing relative differences in mortality by marital status in several countries, but few studies have considered trends in cause-specific mortality by marital status among elderly people.

**Methods:**

The author uses discrete-time hazard regression and register data covering the entire Norwegian population to analyze how associations between marital status and several causes of death have changed for men and women of age 75-89 from 1971-2007. Educational level, region of residence and centrality are included as control variables. There are 804 243 deaths during the 11 102 306 person-years of follow-up.

**Results:**

Relative to married persons, those who are never married, divorced or widowed have significantly higher mortality for most causes of death. The odds of death are highest for divorcees, followed by never married and widowed. Moreover, the excess mortality among the non-married is higher for men than for women, at least in the beginning of the time period. Relative differences in mortality by marital status have increased from 1971-2007. In particular, the excess mortality of the never married women and, to a lesser extent, men has been rising. The widening of the marital status differentials is most pronounced for mortality resulting from circulatory diseases, respiratory diseases (women), other diseases and external deaths (women). Differences in cancer mortality by marital status have been stable over time.

**Conclusions:**

Those who are married may have lower mortality because of protective effects of marriage or selection of healthy individuals into marriage, and the importance of such mechanisms may have changed over time. However, with the available data it is not possible to identify the mechanisms responsible for the increasing relative differences in mortality by marital status in Norway.

## Background

It has been documented several times that those who are married have lower mortality than those who are not, both among elderly people and the younger [1-3]. The few studies investigating how the marital status differentials in old-age mortality have changed over time suggest that excess mortality among the non-married has increased. Murphy et al. [4] report increasing relative differences in mortality by marital status among elderly people aged 60-89 in seven European countries during the 1990s. Valkonen et al. [5] show increasing marital status differences in mortality from 1970 to 1995 in several European countries and Canada for men and women in the age group 65-74. However, the authors of these two studies were not able to control for important confounders such as socioeconomic status. Martikainen et al. [6] also report increasing relative marital status differentials in Finland at ages over 65 for most causes of death in the time period 1976-2000, and control for socioeconomic status and household composition.

Because studies of trends in the relationship between marital status and mortality among elderly people are sparse, and only a small number of them analyze also data on cause-specific mortality, the objective of the present study is to investigate how the associations between marital status and several causes of death have changed for Norwegian males and females of age 75-89 from 1971-2007. Data for the entire Norwegian population will be used, and I will control for the marital status differentials in education and place of residence.

## Methods

The research is based on nation-wide data from different registers and censuses, which have been linked through the personal identification number given to everyone who has lived in Norway after the Norwegian Central Population Register was established in 1964. The data are not available to the public. From the registers there is information on marital status 1st of January every year since 1971 and municipality of residence and dates of in-and out-migration to the country 1st of January every year since 1971. Data on highest level of education achieved as of 1st of October every year is available from the National Education Database since 1980, whereas educational level before 1980 is extracted from the Population Census in 1970. From the Norwegian Causes of Death Register there is information on date and cause of death (if any). Causes of death have been coded according to the International Classification of Diseases (ICD) using the eight revision from 1969-1985, the ninth revision from 1986-1995 and the 10^th ^revision from 1996 [7]. I focus on some main causes of death: colon/rectum cancer, lung cancer, breast cancer, prostate cancer, circulatory diseases, respiratory diseases, other diseases and external causes (table 1). Information on cause of death is missing for less than one percent of the sample and they have been excluded from the cause-specific analysis.

**Table 1:** Causes of death

Cause of death		ICD-10 (1996-)	ICD-9 (1986-1995)	ICD-8 (1969-1985)
Cancers *of which:*		C00-C97	140-208	140-209
	Colon/rectum cancer	C18-C21	153-154	153-154
	Cancer of lung, trachea and bronchus	C32-C34	161-162	161-162
	Breast cancer	C50	174-175	174
	Prostate cancer	C61	185	185
Circulatory diseases		I00-I99	390-459	390-444.1, 444.3-458, 782.4
Respiratory diseases		J00-J99	460-519	460-519
External causes		V01-Y89	E800-E999	E800-E999

Source: [7]

The study is based on discrete-time hazard regression. A series of one-year observations was created for each Norwegian man and woman, starting at age 75 or in 1971 (whatever occurred last) and ending in year 2007, at age 89, at time of death or last emigration (whatever occurred first). For men there were 384 101 deaths during the 4 335 845 person-years of follow-up, while there were 420 142 deaths during the 6 766 461 person-years for women. For each one-year observation different independent variables referring to the situation in the beginning of the year were included. Based on all these observations, a logistic regression model for death probabilities was estimated using the Proc Logistic procedure in SAS version 9.2 [8].

Two procedures were used to analyze the trends in marital status differentials. First models were estimated separately for the years 1971-1979, 1980-1989, 1990-1999 and 2000-2007. Within each time period calendar year and age were included as continuous variables. Moreover, controls for education classified into four groups were included; compulsory education, secondary education, higher education and postgraduate education. Less than one percent of the sample had missing information on education, and were grouped together with persons with compulsory education. Excluding them from the analyses would not affect the results. Life expectancy at birth varies from 76.3 to 79.7 years among men and 81.6 to 84.2 years among women across Norway's 19 counties [9]. Because marriage also is more common in certain regions or in rural rather than urban areas, control variables for region of residence (the 19 counties classified into five main regions) and centrality were included. The variable centrality measures the municipality's geographical location relative to a centre with higher order functions such as bank or post office, as well as related to population size. A person's current municipality of residence is classified into one out of four centrality categories based on Statistics Norway's standard classification.

The second procedure was to check the possibility of a linear trend over the period 1971-2007 by adding interaction effects between marital status and period (operationalized as year minus 1990, so the main effects of marital status could be interpreted as the effects in 1990, which is in the middle of the study period). In these models, also the control variables were interacted with period in order to control for their potential confounding role on the association between marital status and mortality over time. This is a highly relevant issue. For example, differences in mortality by educational level have increased over time, both among middle-aged and elderly Norwegians [10,11].

## Results

Descriptive statistics are presented in table 2. For both sexes, causes of death distributions have changed from 1971 to 2007, with a reduction in deaths from circulatory diseases and an increase in cancer deaths. Mean age has increased from 1971-2007 mainly because of higher life expectancy over time. There has also been a marked change in relationship patterns. Most pronounced is the reduced proportion of never married persons, especially among women (from 20% to 7%). The main reason is low marriage rates for those born in the beginning of the 20^th ^century, while marriage was almost universal from the 1930s to the 1960s [12]. The proportions of divorcees have also increased over time, but this is still a relatively small group in the old age groups in focus here. Moreover, the average educational level has become higher over time, while the regional and centrality distributions have been fairly stable.

**Table 2:** Distribution of Norwegian men and women aged 75-89 from 1971-2007

	Men				Women			
**Time period**	1971-1979	1980-1989	1990-1999	2000-2007	1971-1979	1980-1989	1990-1999	2000-2007
**Number of person-years**	804 394	1 062 962	1 247 666	1 084 196	1 185 901	1 696 600	2 010 678	1 672 275
**Number of deaths**	77 617	99 654	111 268	85 393	86 037	109 305	121 254	92 983
**Crude death rate**	0.10	0.09	0.09	0.08	0.07	0.06	0.06	0.06
**Cause of death (%)**								
Cancers	18.12	20.60	22.89	25.83	14.21	16.63	17.77	20.22
Circulatory diseases	55.08	52.29	49.12	41.54	58.11	55.36	51.32	43.88
Respiratory diseases	11.50	11.30	11.49	11.69	11.54	10.40	10.61	10.16
Other diseases	12.62	12.65	13.24	17.26	12.51	13.68	16.92	22.27
External causes	2.63	3.05	2.95	3.08	3.58	3.83	3.20	3.15
Missing cause of death	0.05	0.11	0.31	0.60	0.05	0.10	0.18	0.32
**Mean age in years (SD)**	79.7 (3.8)	79.9 (3.9)	80.0 (3.9)	80.3 (3.9)	80.0 (3.9)	80.4 (4.0)	80.6 (4.0)	81.1 (4.1)
**Marital status (%)**								
Never married	12.2	11.5	9.7	8.3	20.3	16.5	10.7	7.0
Married	58.7	62.4	65.3	66.6	23.9	24.4	27.2	29.7
Widowed	27.4	24.0	21.8	20.2	53.1	56.1	58.3	58.2
Divorced	1.7	2.1	3.2	4.9	2.8	3.0	3.8	5.1
**Education (%)**								
Compulsory	70.5	63.5	54.4	44.6	76.3	70.1	64.9	56.7
Secondary	22.9	28.6	35.5	40.6	20.0	25.5	30.0	36.0
Higher	4.1	4.5	6.0	9.5	3.5	4.1	4.7	6.8
Postgraduate	2.5	3.4	4.1	5.3	0.2	0.3	0.4	0.5
**Region (%)**								
East	50.5	50.3	49.9	50.6	52.2	52.2	51.2	51.0
South	5.9	5.5	5.6	5.6	5.7	5.4	5.6	5.7
West	24.1	24.7	25.2	24.8	24.2	24.3	24.6	24.5
Central	9.3	9.3	9.2	9.1	8.7	8.7	8.8	8.8
North	10.2	10.2	10.1	9.9	9.2	9.4	9.8	10.0
**Centrality (%)**								
Least central	16.8	16.0	14.6	13.1	13.6	13.2	13.0	12.5
Less central	7.7	7.6	7.5	7.2	6.8	6.7	6.9	6.9
Quite central	19.3	19.3	19.4	19.3	18.1	18.1	18.5	18.8
Central	56.2	57.1	58.5	60.4	61.5	62.0	61.6	61.8

### Trends in mortality by marital status for the years 1971-1979, 1980-1989, 1990-1999 and 2000-2007

**Figure 1:**
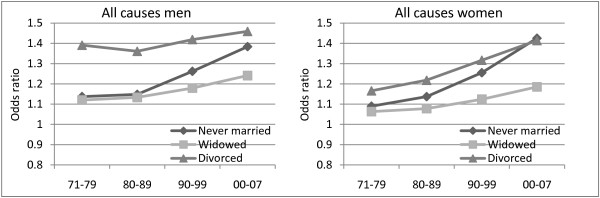
**Trends in all-cause mortality by marital status**. Odds ratios. Norwegian men and women aged 75-89. Reference category is married. Controlling for year, age, level of education, region of residence and centrality. Filled data point = coefficient significant at p < 0.05.

**Figure 2:**
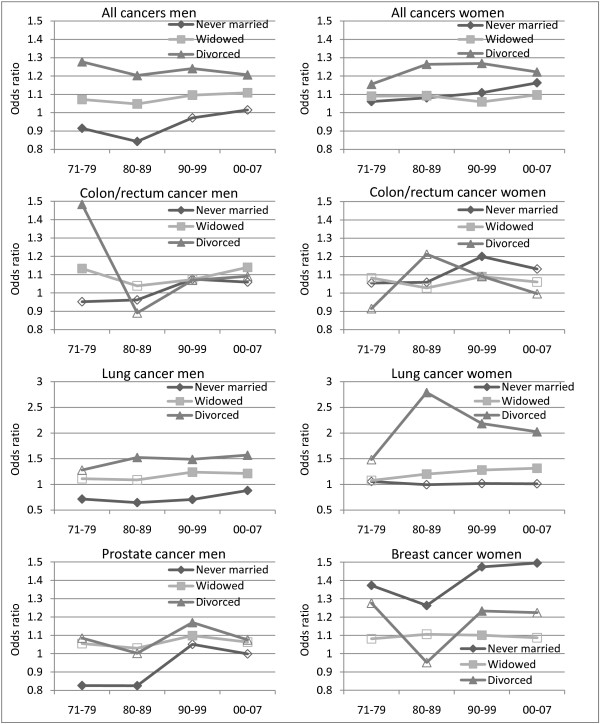
**Trends in mortality from all cancers, colon/rectum, lung, prostate and breast cancer by marital status**. Odds ratios. Norwegian men and women aged 75-89. Reference category is married. Controlling for year, age, level of education, region of residence and centrality. Filled data point = coefficient significant at p < 0.05.

**Figure 3:**
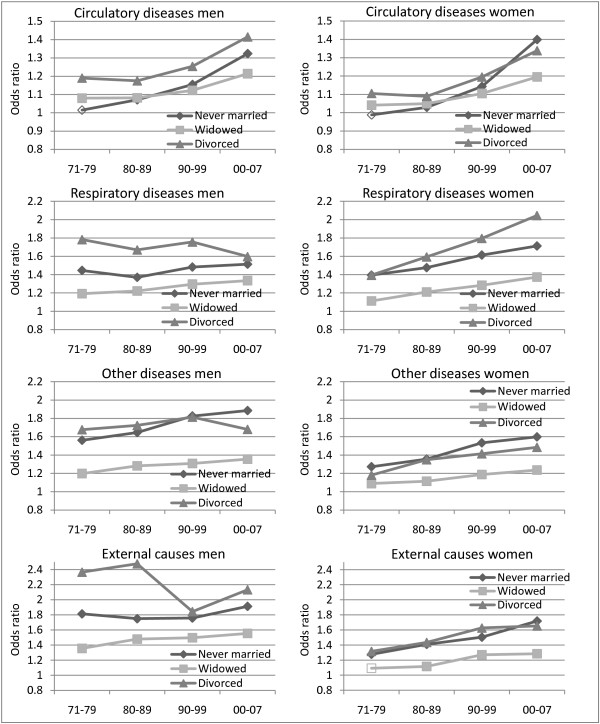
**Trends in mortality from circulatory, respiratory and other diseases and external deaths by marital status**. Odds ratios. Norwegian men and women aged 75-89. Reference category is married. Controlling for year, age, level of education, region of residence and centrality. Filled data point = coefficient significant at p < 0.05.

Figures 1, 2 and 3 show relative differences in mortality by marital status from the selected causes of death for the years 1971-1979, 1980-1989, 1990-1999 and 2000-2007. In all models there are controls for year, age, educational level, region of residence and centrality. There are pronounced differences in all-cause mortality by marital status, particularly for men (Figure 1). The mortality gap is especially large between divorced and married persons. From 1971 to 2007 the excess mortality compared to the married has increased for most groups of non-married, and most clearly for the never married. Moreover, the increase has been more pronounced for women than for men. In the time period 2000-2007, divorced, never married and widowed women had almost as high excess mortality relative to those who were married as their male counterparts.

For all cancers together, marital status differentials have been fairly stable over time (Figure 2). However, never married men have significantly lower risk of dying relative to their married counterparts in the beginning of the study period, and an increasing advantage from 1971-79 to 1980-1989. The pattern varies across cancer types, though (Figure 2). There are few significant marital status differentials in mortality from colon/rectum cancer and no obvious time trends. Apparently there is a marked drop in the excess mortality for divorced men over time. However, their odds of death are not significantly different from that of married men from the 1980s. With respect to lung cancer mortality, never married men are at a significantly lower level than those who are married, whereas divorcees have a significant excess mortality. The gap is more or less stable over time. Differences in lung cancer mortality between divorced and married women have been reduced over time, while there has been a slight increase in the excess mortality of widows. Significant differences in mortality from prostate cancer are only found between never married and married men in the beginning of the study period, where the first group shows a lower risk, while divorcees and widowers have significantly higher mortality levels as compared to married men only in the third time period. The risk of dying from breast cancer is significantly higher among never married women relative to those who are married, and the differences have increased since the 1980s.

There are large and increasing marital status differentials in mortality from circulatory diseases (Figure 3). The increase is most pronounced for ischemic heart diseases, whereas the increase in the marital status differentials in cerebrovascular mortality has been more modest (results not shown).

In general, marital status differences in mortality from respiratory diseases are large (Figure 3). However, time trends are different for males and females. For males, there has been a convergence over time due to lower excess mortality among divorced men, whereas females have experienced increased relative differences in all non-married groups. Large and increasing differences in mortality by marital status are also found for mortality from other diseases and external deaths (Figure 3). The exception is divorced men, for whom there is no clear trend in the excess mortality with respect to external deaths.

### Trends in mortality by marital status for the years 1971-2007, based on models including period interactions

Table 3 shows linear trends in the association between marital status and all-cause mortality (i.e. interactions between marital status and period) for the whole time period 1971-2007. There is a significant increase in excess all-cause mortality for all non-married groups relative to those who are married, with no substantial gender differences, except for the divorced group where the increase is statistically significant only among women. The highest excess mortality is among the never married group, with an increase of 0.8-0.9% per year from 1971 to 2007, respectively for men and women. Significantly increasing mortality differentials are also found for deaths from cancers (an increasing excess mortality for never married males and females and widowed males), circulatory diseases, respiratory diseases (an increasing excess mortality for widowed males and all females), other diseases (except for divorced men) and external causes (an increasing excess mortality for widowed males and females and never married females) (results not shown).

**Table 3:** Trends in all-cause mortality by marital status 1971-2007

	Men	Women
Year^c^	0.938***(0.931-0.944)	0.952***(0.946-0.959)
Married (ref.)^d^	1.000	1.000
Never married	1.230***(1.219-1.241)	1.222***(1.210-1.233)
Widowed	1.168***(1.160-1.176)	1.112***(1.104-1.121)
Divorced	1.406***(1.386-1.426)	1.279***(1.260-1.297)
(Year-1990)*married (ref.)	1.000	1.000
(Year-1990)*never married	1.008***(1.006-1.009)	1.009***(1.008-1.010)
(Year-1990)*widowed	1.004***(1.003-1.005)	1.004***(1.003-1.005)
(Year-1990)*divorced	1.001(0.999-1.003)	1.007***(1.005-1.009)

Odds ratios. Norwegian men and women aged 75-89^ab^

^a^Controlling for age, level of education, region of residence, centrality, age*(year-1990), education*(year-1990), region of residence*(year-1990) and centrality*(year-1990).

^b^*p < 0.05, **p < 0.01, ***p < 0.001

^c^Current year of exposure to death

^d^Main effects of marital status can be interpreted as the effects in 1990.

## Discussion

### Differences in mortality by marital status

The results are similar to those reported in several other studies, which have also concluded that elderly non-married persons have an excess mortality from all-cause mortality [1-6,13,14], cancers [2,14], cardiovascular mortality [2,6,14], respiratory diseases [6] and external deaths [6].

The higher mortality found for most causes of death among elderly non-married Norwegians may be due to protective effects of marriage. First, married persons are likely to benefit from various types of support [15]. A spouse may exert control on behavior, offer practical help, add to the pool of knowledge and help interpreting important information. Second, having a spouse is an economic advantage because of specialization, economies of scale and pooling of wealth [16]. Through either channel, marital status may be associated with various lifestyle factors of importance for the *occurrence *of a number of potential lethal diseases. Examples of such lifestyle factors are smoking, which increases the risk of lung cancer, cardiovascular and respiratory diseases [17], overweight and obesity, which are associated with a high risk of cardiovascular diseases and various cancer types, including breast cancer, colorectal cancer and aggressive prostate cancer [18,19], physical activity which lowers the risks for several diseases, and alcohol consumption, which increases the risk of external deaths, liver diseases and possibly breast cancer [20]. Moreover, some diseases are associated with reproductive factors that are linked with marital status. For example, low sexual activity is associated with low occurrence of prostate cancer [21], and this may be one reason for the lower mortality for this cause of death observed among never married men in the beginning of the study period. Moreover, the high excess mortality from breast cancer found among never married women may be partly due to childlessness as childbearing has a protective effect through physiological mechanisms [20].

Protective effects of marriage may also operate through factors affecting *survival *from different diseases. First, those who are married may get diagnosed and seek treatment earlier, and therefore have a better prognosis [22]. This is especially important in case of cancer, where the results show significant marital status differences in mortality. Second, health status at time of diagnosis and health behavior in the subsequent period are important predictors of survival and related to marital status through the mechanisms mentioned above. Third, those who are married may get better treatment e.g. because their spouse help them to ask for a second opinion, discuss possible treatments and help them follow the given instructions [23].

In addition to being associated with current marital status, mortality may be related to the time since the most recent change in marital status as well as the earlier history. In particular, some of the previously married may have experienced death of the partner quite recently, followed by stress reactions that add to the aforementioned problems associated with being non-married. This may be most relevant for external causes of death, where the results show high excess mortality, especially for men. In support of this kind of explanation, a Finnish study has shown that excess mortality is especially high for accidental and violent causes of death shortly after having become widowed, even among elderly people [24].

Another reason for the marital status differentials in mortality is that certain characteristics affect both mortality and family behavior. Most importantly, poor physical or mental health typically reduce the chance of marrying and remaining married, though the opposite is also possible, because presumed protective effects of marriage may give persons in poor health incentives to marry [25]. Furthermore, there may be a selection into or out of marriage with respect to characteristics affecting health behavior, risk-taking behavior or emotional stability, such as socio-economic status or values [26].

A striking result is the lower risk of dying from lung cancer among elderly never married men through the whole time period. However, this is in accordance with findings from a Dutch study based on the entire population of 25 years and older [27]. Lower lung cancer mortality among never married men may be due to smoking behavior as some studies suggest that the proportions of smokers among the never married are relatively low [28,29]. It is less likely that the lower mortality can be explained by health selection over age, as these patterns also were present in younger age groups (results not shown).

### Trends in mortality by marital status

The results show increasing marital status differences in all-cause mortality for elderly Norwegian males and females. This is in line with findings from other studies focusing both on older people and on younger age groups [3-6,14,30]. I was able to identify only a few studies investigating trends in cause-specific mortality by marital status. In accordance with findings from Israel for the age group 65-89 [14] and the US for ages over 40 [30], but as opposed to the pattern reported from Finland [6], this study did not show any increase in marital status differences in cancer mortality. The results for cardiovascular mortality are similar to those reported from Israel [14], the US [30] and Finland [6], and show increasing differences by marital status over time. In accordance with the findings from Martikainen et al. [6], there were divergent trends in female mortality from respiratory diseases, whereas excess mortality for divorced males decreased over time, leading to an overall reduction in the marital status differences for men. Contrary to the results from the US [30] but similar to those from Finland [6], widening marital status differentials in external deaths were observed. Moreover, the increase in marital status differences in all-cause mortality in the current study was most pronounced for women, and the gender differences in mortality by marital status were almost equal in the end of the study period.

There may be different reasons why associations between marital status and mortality have changed over time among elderly Norwegian men and women. First, the increasing excess mortality among never married persons evident for most causes of death may indicate that they receive less support from others as the proportion never married, among whom they perhaps are most likely to have their closest companions, has become smaller. Social support from persons sharing the same situation may be most important for never married persons, as divorcees and widows/widowers are more likely to have children. Moreover, there is probably a stronger focus on self-realization today, and people may care most for their closest family. This may have played a role for the reported increase in excess mortality among the non-married in general. Second, the economic benefits of marriage were mostly due to specialization earlier-with the female partner having responsibility for home and child care while the man was the only wage earner [16]. It has been suggested that the economic benefit now to larger extent lies in the pooling of resources and advantages of scale e.g. [31], and one may speculate whether this perhaps is less of an advantage. The literature is not conclusive on this topic, but some report lower economic gains from marriage over time, at least for men [32]. However, any such change may be of modest relevance for the cohorts in focus of the current study, among whom women's labor force participation has still been low. Third, it may have become more accepted and thus less stressful, to be divorced as this status has become more common [33]. This may suggest why the disadvantage for divorced men has been stable over time, and even reduced for some causes of death. Fourth, there may be changes in selection into and out of marriage over time. Some studies have focused on changes in relative group size and argue that decreasing shares of non-married persons indicate stronger selection with respect to health and cultural or socioeconomic characteristics that have a bearing on health e.g. [34]. This may be the case for never married elderly people. Their relative group size has decreased from 1971-2007, whereas their excess mortality relative to those who are married has increased. However, changing proportions in the non-married and married population is a too vague indication of the changing direction of the selection. The important issue is whether characteristics that have a negative effect on health or health behavior have become more or less important for marriage formation and dissolution. Unfortunately, there is little knowledge about this. For example, we do not know whether it has been considered increasingly important to find a partner who has good health or a high wage potential, or who is a good problem-solver, all of which would be potentially negatively linked to later mortality. In particular, the relative-size argument is irrelevant for the widowed, because a smaller group of widowers is due to increasing life expectancy over time and changing sex differentials in mortality.

Even though several causes of death have common risk factors, investigating trends in cause-specific mortality may indicate how associations between marital status and risk factors for different causes of death have changed over time. For example, increasing excess mortality from circulatory diseases among the non-married may be due to changing diet more than changing smoking patterns, as there is no increase in marital status differentials in lung cancer mortality. According to one Swedish and one Finnish study, healthy diet became more common among married than non-married women over time [35,36].

There are some limitations in the current study. First, interpreting trends in cause-specific mortality is in principle problematic because of changes in ICD revisions and coding practices over time. However, this is less reason for concern when the focus is on broad causes of death like here. Second, information on cause of death from death certificates is less reliable among elderly people [37]. This is particularly the result of co-morbidity, which is especially common among older persons and perhaps above all those with low levels of social support [38]. In other words, cause of death may be most inaccurate in the non-married population, and can bias the estimates for these groups. Third, the only socioeconomic factor controlled for in this analysis is the educational level, which is relatively homogenous in the age groups in focus. It has been suggested that socioeconomic status among elderly people also should be measured with income and former occupation to give a more comprehensive picture [39]. Fourth, there is no information on cohabitation status as far as back to 1971 in the Norwegian registers. Those living with a partner, friend or relative without being married are registered in one of the non-married groups. When data on cohabitation status is lacking and this group is large, differences in mortality by marital status would be underestimated, as the mortality e.g. among those living with a partner without formal marriage is lower than among other non-married groups [40]. However, there are few persons living with a partner without a formal marriage in the old age groups in focus in the present study. Survey data from Statistics Norway shows that this group constitute less than two percent in the age group 70-79 from 1993-2007 [41].

Despite some limitations, this study contributes to the debate on differentials and trends in mortality by marital status because of the long study period, the large data material and the focus on cause-specific mortality. The reported increase in excess mortality among never married, divorced and widowed persons should be monitored as this points towards a large demand for health care. Moreover, the results from the cause-specific analyses indicate that there is a need to investigate whether marital status differentials in risk factors associated with circulatory diseases, respiratory diseases and external deaths, in which the increase in excess mortality among non-married was most pronounced, have changed over time.

## Conclusions

This study, which is based on large data of high quality, confirms that differences in mortality by marital status are present among elderly Norwegian males and females, and for most causes of death. In addition, relative differences in mortality by marital status have increased over time. The largest marital status differences in mortality are found for deaths from respiratory diseases, other diseases and external causes, whereas the largest increases in marital mortality differences are found for mortality from circulatory diseases, respiratory diseases, other diseases and external deaths. For cancer mortality marital status differentials have been stable over time.

## Competing interests

The author declares that they have no competing interests.

## Authors' contributions

KNB conceived of the study, performed the design and the statistical analysis, drafted the paper and approved the final manuscript.

## Acknowledgements

The author would like to thank Øystein Kravdal and four reviewers for very helpful comments, and appreciates financial support from the Research Council of Norway.

## Pre-publication history

The pre-publication history for this paper can be accessed here:

http://www.biomedcentral.com/1471-2458/11/537/prepub
